# Editorial: Neuronopathic lysosomal storage diseases - specific neuronal characteristics and therapeutic approaches

**DOI:** 10.3389/fnmol.2022.1078804

**Published:** 2022-11-08

**Authors:** Stefanie Flunkert, Birgit Hutter-Paier, Ying Sun, Jan Kehr

**Affiliations:** ^1^QPS Austria, Grambach, Austria; ^2^BioDoks e.U., St. Georgen an der Stiefing, Austria; ^3^Division of Human Genetics, Cincinnati Children's Hospital Medical Center, Cincinnati, OH, United States; ^4^Department of Pediatrics, University of Cincinnati College of Medicine, Cincinnati, OH, United States; ^5^Department of Pharmaceutical Biosciences, Uppsala University, Uppsala, Sweden; ^6^Pronexus Analytical SA, Bromma, Sweden

**Keywords:** animal models, neuronopathic Gaucher disease, mTORC1, TFEB, Krabbe disease, umbilical cord blood transplantation (UCBT)

Treatment of neuronopathic lysosomal storage diseases (LSD) represents an exceptional challenge for the research community, as a substance does not only have to be active in the periphery but needs also be able to cross the blood-brain barrier in order to act on neurological symptoms. The most promising approaches to treat LSDs consistently are currently gene therapy methods. To promote the development of new treatments against LSDs, several topics are of utmost relevance: The cause of each LSD and their pathophysiology observed in patients need to be evaluated to be able to create treatments that can cure the disease or at least ameliorate symptoms to a tolerable level. Such results will further support the development of *in vitro* and *in vivo* animal models with high predictive, face, and construct validity. These models will then allow LSD research beyond the patient and will additionally also support the development of new treatments by providing a platform for efficacy studies outside the patient.

Our Research Topic thus welcomed manuscripts that provide valuable insights into the pathophysiology of LSD patients, new and existing research models of neuronopathic LSDs, as well as related drug development studies. Six manuscripts were submitted to the Research Topic. Eventually, 4 manuscripts were accepted after the peer-review process, two manuscripts covering research about the relevance of microglia and different cell organelles for Gaucher disease and two manuscripts introducing new animal models and describing the impact of umbilical cord blood transplantation on Krabbe disease.

## Gaucher disease

### Importance of microgliosis in neuronopathic Gaucher disease

Zhang et al. investigated differential activation of neuroinflammatory mediators in relation to dysfunction in mTORC1 activity and the association to lysosome function in microglia, neurons and astrocytes in 4L;C^*^ mice, a genetic model representing a murine model of neuronopathic Gaucher disease (nGD). The authors found a two-fold increase of microglia in nGD mice. They implemented a novel method for quantifying the transmembrane protein 119 (Tmem119), proposed to act as a specific marker for resident microglia. In addition, microglia showed hyperactive mTORC1 signaling coinciding with increased lysosome function, whereas the neurons showed hypoactive mTORC1 function without consistent alteration in lysosome function and the reactive astrocytes displayed only neglectable changes. These findings highlight the differences and complexity of molecular mechanisms that are involved in nGD pathogenesis within these three major types of brain cells.

### Organelle dysfunctions beyond lysosomes in neuronopathic Gaucher disease

Arévalo et al. provide a comprehensive review article about cell organelles that are affected by dysfunctional lysosomes in neuronal Gaucher disease. To start with, authors list existing cellular and animal models of Gaucher disease and introduce the most relevant signaling pathways for organelle dysfunctions; authors focus on the lysosomal master transcription factor TFEB and the kinases RIPK1 and RIPK3 that are involved in necroptotic cell death. Afterwards, authors summarize the effects of dysfunctional lysosomes on the endoplasmic reticulum, autophagosomes and mitochondria. Lastly, results about the GOLGI complex and exosomes are mentioned, although not many data exist about the impact of lysosomal dysfunction on these organelles in Gaucher disease.

## Krabbe disease

### Novel early- and adult-onset Krabbe disease mouse models

Rebiai et al. developed two new Krabbe disease mouse models by CRISPR-Cas9 Knock-In. By introducing point mutations that are known in humans to cause infantile and adult forms of Krabbe disease, authors were able to generate transgenic mouse models that closely mimic these two forms of the disease. Authors provide a rigorous characterization of both models. The model of infantile Krabbe disease presents a strongly reduced life span, decreased β-galactosyl-ceramidase (GALC) activity and increased psychosine levels as wells as a severe and lethal progression of the neurological phenotype while the model of adult Krabbe disease shows an unaltered survival rate, and only slightly changed GALC activity and psychosine accumulation levels. The latter model is instead characterized by mild inflammatory demyelination and deficits in coordination, motor skills and memory that develop with age. As these two mouse models are based on genetic alterations that are indeed observed in Krabbe disease patients accompanied by corresponding neuropathological phenotypes, they should quickly replace the *twitcher* mouse as first choice model in Krabbe disease research.

### Umbilical cord blood transplantation to improve brain pathology in infantile Krabbe disease

Kofler et al. reported the pathological findings of post-mortem examinations of two patients with infantile Krabbe disease. One patient received umbilical cord blood transplantation at 4 weeks of age which extended lifespan to 15 years. The second patient left untreated passed at the age of 7 years. MRI scans and the neurocognitive development score revealed stabilized white matter disease and delayed functionality decline in the transplanted patient compared to the untreated patient. By microscopic examination, the transplantation improved myelination, preserved neurons and resulted in absence of globoid cells in the brain and spinal cord. However, umbilical cord blood transplantation had minimal benefits on the peripheral nervous system. Autopsy findings in progressive neurological lysosomal diseases are scarce and exceptionally valuable. This study is a significant contribution to the understanding of the neuropathology of Krabbe disease, especially the beneficial outcome in hindering the disease progression by umbilical cord blood transplantation.

## Author contributions

SF, YS, and JK wrote the manuscript. All authors edited and approved the manuscript.

## Conflict of interest

Authors SF and BH-P were employed by QPS Austria GmbH. Author SF is owner of BioDoks e.U. Author JK was employed by Pronexus Analytical SA. The remaining author declares that the research was conducted in the absence of any commercial or financial relationships that could be construed as a potential conflict of interest.

## Publisher's note

All claims expressed in this article are solely those of the authors and do not necessarily represent those of their affiliated organizations, or those of the publisher, the editors and the reviewers. Any product that may be evaluated in this article, or claim that may be made by its manufacturer, is not guaranteed or endorsed by the publisher.

